# Association Mechanism and Conformational Changes in Trypsin on Its Interaction with Atrazine: A Multi- Spectroscopic and Biochemical Study with Computational Approach

**DOI:** 10.3390/ijms23105636

**Published:** 2022-05-18

**Authors:** Arwa Ishaq A. Khayyat, Seema Zargar, Tanveer A. Wani, Muneeb U. Rehman, Azmat Ali Khan

**Affiliations:** 1 Department of Biochemistry, College of Science, King Saud University, Riyadh 11451, Saudi Arabia; aalkhyyat@ksu.edu.sa; 2 Department of Pharmaceutical Chemistry, College of Pharmacy, King Saud University, Riyadh 11451, Saudi Arabia; azkhan@ksu.edu.sa; 3 Department of Clinical Pharmacy, College of Pharmacy, King Saud University, Riyadh 11451, Saudi Arabia; muneebjh@gmail.com

**Keywords:** atrazine, trypsin, activity, fluorescence, quenching, docking

## Abstract

Atrazine (ATR) is a herbicide globally used to eliminate undesired weeds. Herbicide usage leads to various adverse effects on human health and the environment. The primary source of herbicides in humans is the food laced with the herbicides. The ATR binding to trypsin (TYP) was investigated in this study to explore its binding potential and toxicity. In vitro interaction of ATR with TYP was studied using multi-spectroscopic methods, molecular docking, and enzyme kinetics to explore the mechanism of binding for the TYP-ATR system. The TYP-ATR complex revealed binding constants (10^3^ M^−1^), suggesting a moderate binding. The free energy for the TYP-ATR complexes was negative, suggesting a spontaneous interaction. Thermodynamic parameters enthalpy (ΔH) and entropy (ΔS) obtained positive values for the TYP-ATR system suggesting hydrophobic interactions in the binding process. Micro-environmental and conformational changes in TYP molecules were induced on interaction with ATR. Reduced catalytic activity of TYP was observed after interaction with ATR owing to the changes in the secondary structure of the TYP.

## 1. Introduction

**Figure 1:**
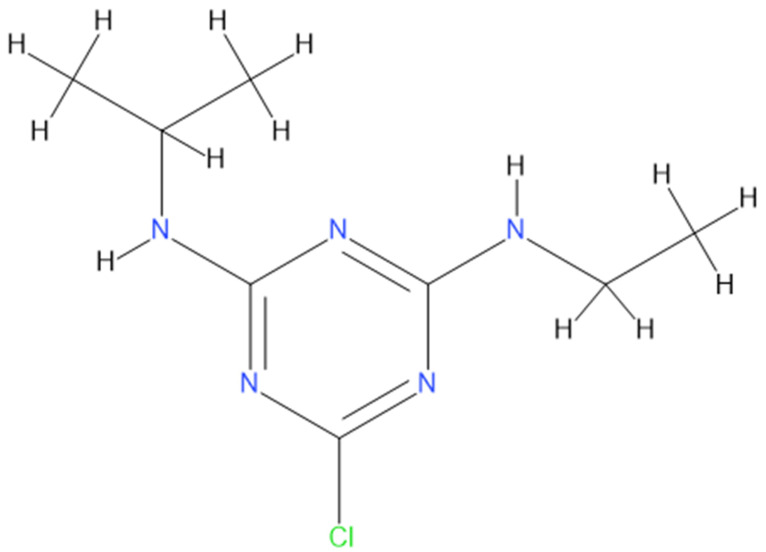
Chemical Structure of ATR.

Herbicide usage is widely practiced in crops to eliminate unwanted plants and weeds, thus essential in food production sustainability [1,2]. However, herbicides are highly toxic chemicals to all organisms, resulting in adverse effects on humans and the environment since they persist in nature for more extended periods [3,4,5,6]. Chemically atrazine (ATR) is 2-chloro-4-ethylamine-6-isopropylamine-1,3,5-triazine (Figure 1) and belongs to the triazine family. An estimated 5.6 billion pounds of herbicides are used worldwide annually [7]. The ATR usage increases production by controlling broadleaf and grassy weeds [8,9]. ATR is highly persistent in the environment and has a half-life of 100 days in surface water. It is commonly detected in contaminated groundwater and waterways [10,11]. Therefore, long-term exposure to ATR can cause adverse effects on human health [12]. Several animal studies confirm that ATZ has endocrine-disrupting ability across multiple animal classes [13,14]. However, few studies reported that ATR inhibits the specific binding of various types of protein receptors (estrogens and progesterone) and therefore interferes with the reproductive health of animals [15,16]. ATZ exposure is also reported to cause epigenetic alterations in DNA methylation and microRNA activity [17,18].

In recent years, proteins have been the main investigational target to study the toxicity of environmental pollutants, especially pesticides, which induce changes in the protein’s structural (conformation) and biological function [19,20,21]. Proteases are essential proteins that play various roles (catalytic ability during physiological and pathological processes) in different biologically relevant processes [22,23,24,25]. Trypsin (TYP), a 23.3 kDa protein containing 223 amino acids, 4-tryptophan, 10-tyrosine, and 6-phenylalanine, is a vital protease (serine-endopeptidase) in the digestive system (pancreas of all vertebrates). Trypsin is essential for multifarious biological functions, including digestion and deconstruction of food proteins and other essential physiological processes [22,23,24]. The catalytic activity and conformational changes of TYP are affected by exogenous environmental pollutants that cause pathological alterations in humans [26,27]. Since no study has been conducted on TYP interaction with ATR, we investigated the interaction mechanism between them and evaluated the conformational changes in TYP. This study provided an insight into the toxicity mechanism of ATR at the molecular level. The influence of ATR on the conformations and catalytic activities of TYP was also explored in this study. Spectroscopic techniques, molecular modeling, and TYP enzyme activity experiments were conducted to comprehend the toxicity mechanism of ATR on the TYP.

## 2. Materials and Methods

### 2.1. Materials

Trypsin (bovine pancreas) ATR, N-α-Benzoyl-D, L-arginine-P-nitroanilide hydrochloride in (BApNA) were all obtained from Sigma-Aldric Co. (St. Louis, MO, USA). Analytical grade solvent and chemicals purchased locally were used in the study.

### 2.2. Sample Preparation

The TYP solution (0.25 mM) was prepared in phosphate-buffered saline (0.1 M containing dibasic (Na_2_HPO_4_), monobasic (NaH_2_PO_4_), and NaCl) stock solution of ATR (0.5 mM) in anhydrous methanol and BApNA (3 mM) was prepared in DMSO. All the reagents were stored at 2–8 °C until use. Buffers were prepared in ultra-pure water.

### 2.3. Instrumentations

The UV-vis absorption spectra were recorded with a UV-1800 double beam spectrophotometer (Shimadzu, Kyoto, Japan). The fluorescence measurements were recorded with an RF-5301PC spectrofluorometer (Shimadzu, Kyoto, Japan). The circular dichroism (CD) measurements were recorded on a JASCO J-1500CD spectrophotometer. FT-IR measurements were carried out on Avatar 360 E.S.P. FT-IR spectrometer (Thermo, Montral-Est, QC, Canada)

### 2.4. Methods

#### 2.4.1. UV-Vis Spectral Measurements

The UV-vis spectra of TYP were recorded in the presence of ATR (0–30 µM). Experiments were performed at fixed TYP concentration (10 µM).

#### 2.4.2. Steady-State Fluorescence Spectroscopy

Fluorescence emission spectra of TYP (10 μM) titrated against ATR (0–45 µM) at 298, 303, and 308 K were recorded. The emission wavelength range was set to 300–500 nm after excitation at 280 nm.

#### 2.4.3. Synchronous Fluorescence Spectroscopy (SFS) Experiments

Synchronous fluorescence spectra measurements of TYP (10 μM) titrated with increasing concentrations of ATR (0–45 µM) were recorded in separate experiments by setting wavelength interval (Δλ) at 15 nm (for tyrosine residues (Y)) and 60 nm (for tryptophan residues (w)) in the same experimental conditions as the steady-state fluorescence emission spectra.

#### 2.4.4. Circular Dichroism (CD) Spectroscopy Measurements

The CD spectra of TYP (5 μM) with ATR (0 and 10 μM) were recorded from 190–260 nm with a scan speed of 20 nm min^−1^ and 1 nm bandwidth. The recorded spectrum was analyzed by the web-based software package tool, CAPITO-CD Analysis and Plotting Tool (uni-jena.de, accessed on 10 April 2022), to evaluate different secondary structures of TYP after being treated with ATR.

#### 2.4.5. Fourier Transform Infrared Spectroscopic (FTIR) Analysis

The FTIR spectra for TYP were recorded with ATR. The FTIR spectroscopy of free TYP (20 μM) and TYP (20 μM)-ATR (100 μM) were recorded in the range of 1800–1400 cm^−1^. The corresponding absorbance of buffer and ATR solutions recorded under similar conditions was subtracted from the spectra.

#### 2.4.6. Computational Analysis

The molecular structure of TYP (PDB ID: 2ZQ1) was obtained from Protein Data Bank (RCSB), http://www.rcsb.org/pdb, accessed on 3 March 2022). Molecular modeling of the ATR−TYP complexation was performed using AutoDock Vina. For docking purposes, water, metal ions, and the initially bound ligand were removed, and hydrogen atoms were added during protein preparation. The pdb structure of ATR was obtained from Pubchem (ID: 2256), and its energy-minimized conformation was analyzed using Chem3D Pro14.0. During the docking study, a grid box defined to enclose the entire binding site of trypsin with dimensions of 40 Å × 40 Å × 40 Å and a grid spacing of 0.375 Å was used. The grid center of the TYP-ATR system was set at −26.319, 1.973, and 16.726. The docked structure of the TYP-ATR system was analyzed with Discovery studio 4.5.

#### 2.4.7. Measurement of Trypsin Activity

The activity of TYP was measured using BApNA as a substrate according to the reported literature [28,29,30]. The absorbance at 400 nm was recorded to evaluate the TYP activity. The relative activity of TYP was expressed as the ratio of activity with and without ATR. The kinetic parameters were determined using a fixed concentration of TYP (5 µM), and the ratios for TYP: ATR were (1:0, 1:5, 1:10, 1:15, and 1:20), BApNA (0–3000 µM), The Michaelis-Menten equation was used in the determination of enzyme reaction velocity [29].

$$V=V_{\max}\frac{\left[S\right]}{K_{m}+\left[S\right]}$$

(1) $$K_{\mathrm{cat}}=\frac{V_{\max}}{\left[E\right]}$$

The above equations, V and V_max_, denote the initial and final reaction velocities, K_m_ is Michaelis Constant, and K_cat_ is the catalytic rate constant. Whereas [E] represents enzyme concentration and [S]: substrate concentration (BApNA). Lineweaver-Burk plots for 1/V vs. 1/[S] at different BApNA concentrations in the absence and presence of ATR.

$$\frac{1}{V}=\frac{K_{m}}{V_{\max}+\left[S\right]}+\frac{1}{V_{\max}}$$

## 3. Results and Discussion

### 3.1. UV-Vis Absorption Spectroscopy Investigations

**Figure 2:**
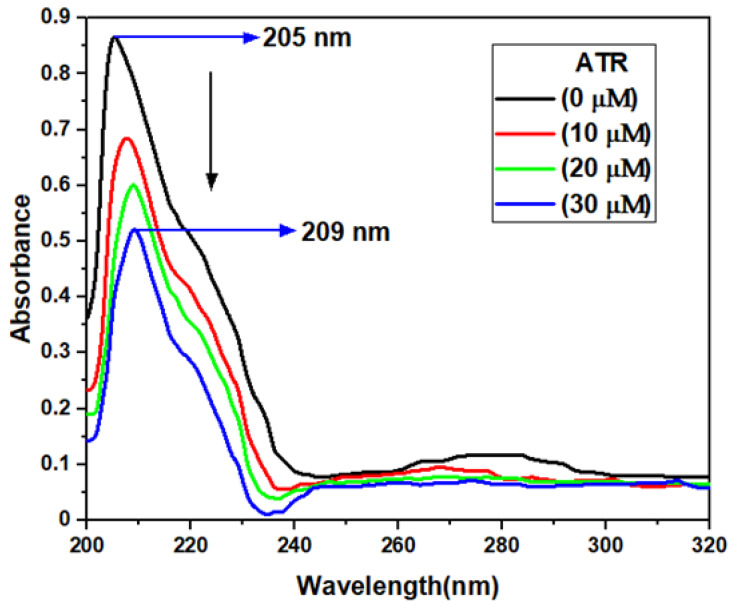
UV-vis absorbance spectra of TYP (10 μM) in presence of increasing concentrations of ATR (0–30 μM).

UV-vis absorption spectroscopy is a simple and versatile technique for studying the conformational and structural changes in a protein and also explores the protein-ligand complex formation [31]. The UV-vis absorption spectra of TYP and TYP-ATR are given in Figure 2. The TYP usually shows two absorption bands, at around 205 nm, and 275 nm. The peak at 205 nm corresponds to peptide bond absorption (secondary structures), and the one at 275 nm represents aromatic amino acids, respectively [32]. A sharp decline in the peak at 205 nm suggests that ATR and TYP interaction might influence the conformation of TYP. The peak at (205 nm) is mainly due to π-π* transitions and corresponds to the polypeptide backbone structure (C=O) of the TYP [33]. Further, a shift in the peak at 205 nm suggests a new complex formation between TYP and ATR. A decline in the absorption at 275 nm also suggests a complex formation occurred between TYP and ATR.

Moreover, the absorbance intensity at 275 nm also decreased, which confirms that the polarity of the microenvironment around the aromatic amino acid residues was affected by the ATR presence [33]. Therefore, the change in absorption peaks and red-shift suggest a conformation change in TYP and complex formation between TYP-ATR and hence a change in the protein structure.

### 3.2. Fluorescence Emission Spectroscopy of TYP-ATR Complex

**Figure 3:**
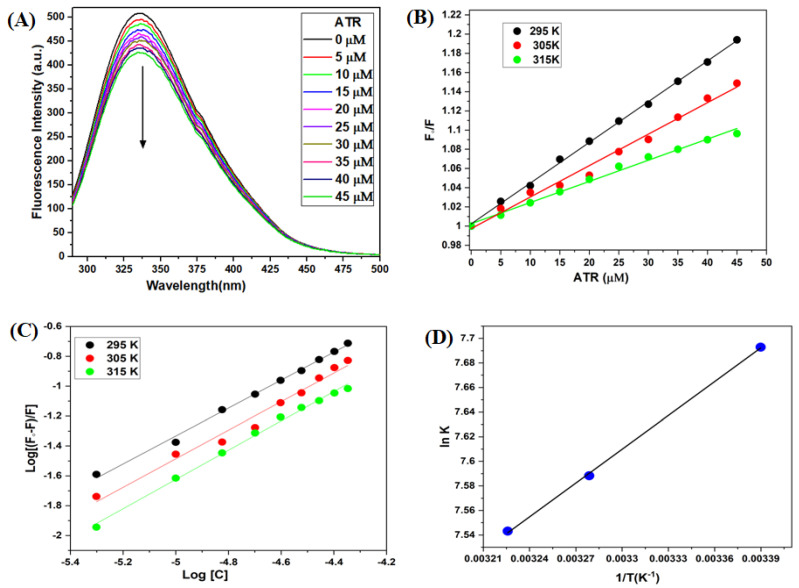
(**A**) Fluorescence emission spectra of TYP (10 μM) in the presence ATR (0–45 μM). (**B**) Stern-Volmer plot for TYP with ATR (0–45 μM) at different temperatures. (**C**) The double-logarithmic plot of [log (F_0_ − F)/F] versus log[C] for TYP-ATR system. (**D**) van’t Hoff plots for TYP-ATR system at different temperatures.

Fluorescence emission spectroscopy is a technique to study the protein-ligand interactions and conformational alterations [34,35]. In the steady-state fluorescence measurements (Figure 3A), TYP exhibited a strong emission peak at 337 nm after excitation at 280 nm. The intrinsic fluorescence of TYP reduced progressively with the addition and increase in ATR concentration. This perception demonstrated a strong binding in the TYP-ATR system and microenvironmental changes in TYP on treatment with ATR [33].

#### 3.2.1. Fluorescence Quenching Mechanism

The fluorescence quenching mechanisms of protein are classified into three mechanisms: static, dynamic, and mixed (static and dynamic) quenching [36]. The quenching mechanism is identified based on their temperature dependence. The fluorescence spectra for the TYP-ATR system were obtained at 295, 305, and 315 K, and the fluorescence data were analyzed with Stern-Volmer equation [37,38]:

$$\frac{F_{0}}{F}=1+K_{\mathrm{sv}}\left[Q\right]=1+k_{q}\tau_{0}\left[Q\right]$$

where F_0_ represents the steady-state fluorescence intensity of TYP, F fluorescence intensity of TYP in the presence of ATR, K_SV_ is the Stern-Volmer quenching constant, and [Q] is the quencher concentration.

The Stern-Volmer plot (Figure 3B) for the TYP-ATR system at (295, 305, and 315 K) shows a decline in the (Table 1) for the TYP-ATR system with temperature rise. This behavior of the K_SV_ values with respect to temperature represents the static quenching mechanism for the TYP-ATR system [37,39].

The bimolecular quenching constant (k_q_) also helps to confirm the quenching mechanism. The k_q_ was determined by [37,40]:

$$k_{q}=K_{\mathrm{sv}}/\tau_{0}$$

where K_SV_ is the Stern-Volmer constant; τ_0_ represents the lifetime (average) of fluorophore without the quencher and has a value of 10^−8^ s [37]. The k_q_ for the TYP-ATR system at different temperatures is presented in Table 1. The values of k_q_ were found higher than the value of the collision quenching constant (2 × 10^10^ M^−1^ s^−1^), suggestive of a static quenching mechanism in the TYP-ATR system [41,42].

#### 3.2.2. Binding Constant Evaluation 

The binding parameters of the TYP-ATR complex, binding constant (K_b_), and binding stoichiometry (n) were evaluated by the equation:

$$\log\frac{\left(F_{0}-F\right)}{F}={\log\;K}_{b}+n\;\log\left[Q\right]$$

where F_0_ and F are the fluorescence intensities of TYP in the absence and presence of ATR, respectively. The K_b_ and n were determined from the plot between the log (F_0_ − F)/F versus log[Q] (Figure 3C, Table 2). The binding stoichiometry n = 1 suggests a single class of available binding sites. The binding constants of >10^3^ present a moderate binding between TYP and ATR. Further, the binding constants decreased with a temperature rise.

#### 3.2.3. Determination of the Binding Forces between TYP and ATR

The intermolecular acting forces, such as hydrophobic, electrostatic interactions, hydrogen bonds, and van der Waals interactions, exist between ligands and macromolecules [43]. The thermodynamic analysis provides evidence about the binding force involved in the interaction. The enthalpy (ΔH°) and entropy (ΔS°) are obtained from the slope and intercept of the va not Hoff plot (Figure 3D) using va not Hoff Equation [39]:

$${\mathrm{lnK}}_{b}=-\frac{\Delta H^{{}^{\circ}}}{\mathrm{RT}}+\frac{\Delta S^{{}^{\circ}}}{R}$$

$$\Delta G^{{}^{\circ}}=\Delta H^{{}^{\circ}}-T\Delta S^{{}^{\circ}}$$

where ∆G° is the binding free energy, R is the gas constant (8.314 Jmol^−1^K^−1^), T represents different temperatures in °K (295, 305, and 315 K), and K_b_ is the binding constant. The calculated values for ΔH°, ΔS°, and ΔG° of the TYP-ATR system are given in Table 3. Further, the negative value for ΔG° confirms the interaction to be spontaneous, and the positive ΔH° and ΔS° values indicate hydrophobic interactions exist in the ATR-TYP system [43,44].

#### 3.2.4. Synchronous Fluorescence Spectroscopy (SFS) Experiment

**Figure 4:**
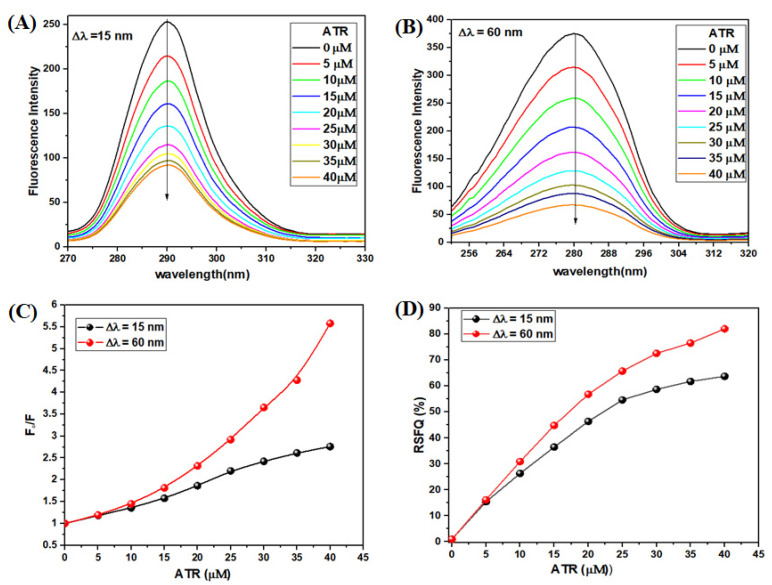
Synchronous fluorescence spectra of TYP (10 μM) with ATR (0–45 μM) at (**A**) Δλ = 15 nm, (**B**) Δλ = 60 nm, pH = 7.4. (**C**) The quenching of TYP synchronous fluorescence of TYP-ATR system. (**D**) Comparison of the effect of ATR on the RSFQ of TYP.

Synchronous fluorescence spectroscopy (SFS) is used to monitor alteration in the microenvironment of fluorophore amino acid residues [45]. The synchronous fluorescence spectra were obtained at Δλ = 15 nm and Δλ=60 nm [46]. The SFS emission spectra of TYP (10 μM) in the presence of ATR (0–45 μM) are given in Figure 4A,B for tyrosine and tryptophan residues, respectively.

The intensity of fluorescence of TYP (around tyrosine residue) decreased in the presence of ATR, and no shift in emission wavelength, confirming no change in the microenvironment around tyrosine residue (Figure 4A). Further, the fluorescence intensity of TYP (around tryptophan residues) decreased significantly in the presence of ATR with a redshift of 1 nm in the emission wavelength. The redshift is suggestive of an alteration in the microenvironment of tryptophan residues. It is also apparent from Figure 4C that the synchronous fluorescence spectra for both tyrosine and tryptophan are significantly different. Further, the ratios of synchronous fluorescence quenching (RSFQ) were estimated by Equation:

$$\mathrm{RSFQ}\left(\%\right)=1-\frac{F}{F_{0}}$$

where F_0_ and F are fluorescence intensities of TYP in the absence and presence of ATR, respectively. To confirm the binding position of the ATR on the surface of TYP, the RSFQ ratio for the TYP-ATR system at tyrosine (Δλ = 15 nm) and tryptophan (Δλ = 60 nm), respectively, were determined according to the above equation (Figure 4D). As a result, the synchronous fluorescence of tryptophan residue is strongly quenched (82% reduction), whereas, in the case of tyrosine residue, the quenching was about 63% in the presence of ATR. Thus, SFS results suggest that ATR affects the microenvironment of the tryptophan residue more than tyrosine on interaction with TYP [47,48].

#### 3.2.5. Circular Dichroism (CD) Spectra Changes in TYP upon ATR Binding

**Figure 5:**
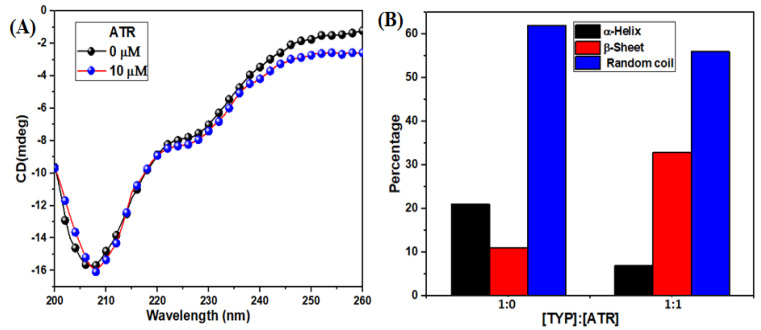
(**A**) The CD spectra of TYP in the presence ATR, and the molar ratio of ATR to TYP were 0:1, 1:1, TYP (10 μM), ATR (10 μM), respectively. (**B**) Relative percentages of the secondary structure of TYP-ATR system.

The CD is a sensitive and powerful technique that explores the protein’s secondary structure, stability, and conformation. It provided information about perturbations in the secondary structure of TYP after treatment with ATR [49]. The spectra of TYP with or without ATR at room temperature (Figure 5A) show a negative peak at 208 nm (π→π* transition), which represents the α-helical structure in TYP [50]. Moreover, addition of ATR decreased the ellipticity of TYP, indicating loss of α-helical contents. The CAPITO-CD analysis software calculated the relative percentages of the secondary structural elements of TYP. The relative percentage of secondary structural elements of TYP (α-helix (21%), β-sheet (11%) and random coil (62%)) and TYP-ART system α-helix (7%), β-sheet (33%) and random coil (56%) (Figure 5B). Therefore, ATR induced changes in the secondary structure of TYP, which may cause a change in the physiological function of TYP-like enzyme activity [51].

#### 3.2.6. FT-IR Spectroscopy

**Figure 6:**
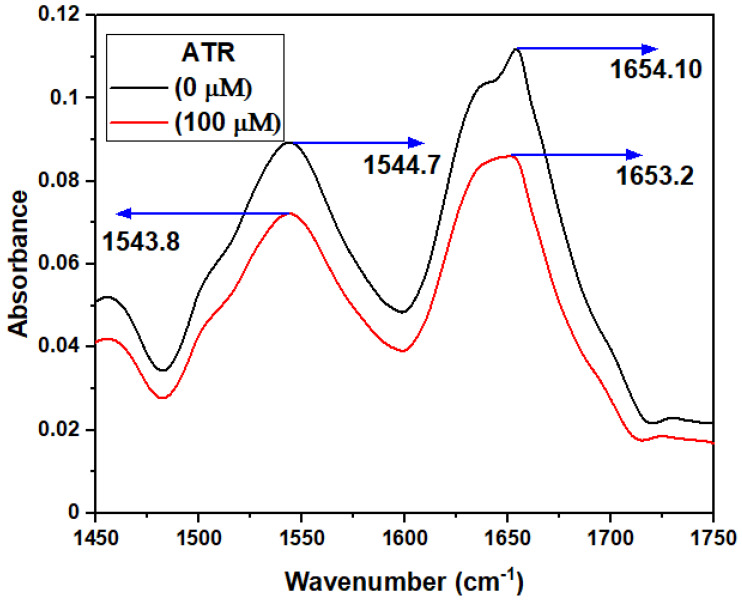
FTIR spectra of TYP (20 μM)-ATR system. The ATR concentrations were 0 and 100 μM, respectively, for the two spectra.

FT-IR spectroscopy is an efficient technique used in detecting changes in the secondary structure of the protein [46]. Proteins exhibit several amide bands (Amide I band (1700–1600 cm^−1^ for C=O) and (Amide II band (1510–1580 cm^−1^ for C-N/N-H vibration)), which represents the secondary structure of TYP [46]. The FTIR spectra of TYP in the absence and presence of ATR (Figure 6) show that the peak position of the amide band shifted slightly from 1654 to1653 cm^−1^ (Amide I) and 1544 to 1543 cm^−1^ (Amide II) in the ATR-TYP complexes, respectively. Thus, TYP interaction with amide I and II groups disturb the naturally occurring conformation and secondary structure of TYP [52]. Although the shift in the peak position is minimal, an alteration in the secondary structure of the protein cannot be ruled out.

#### 3.2.7. Computational Modeling of the TYP-ATR Complex

**Figure 7:**
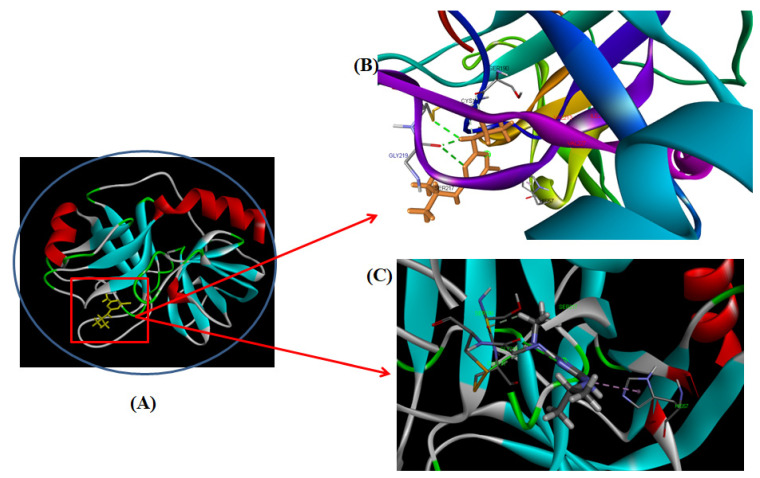
(**A**) Molecular models of TYP complex with ATR. (**B**,**C**) provides a detailed view of the docking poses of the TYP-ATR complex.

**Figure 8:**
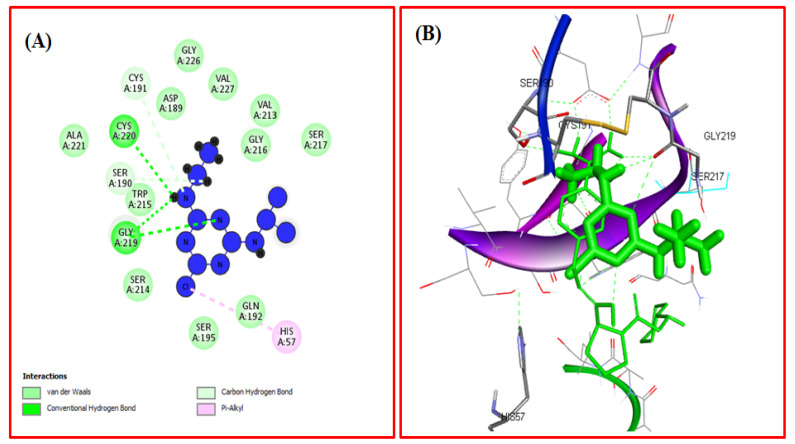
(**A**) The two-dimensional image of the TYP-ATR system and the amino acid residue surrounding the binding pocket. (**B**) Three-dimensional structure of interaction of TYP with ATR.

Molecular docking is a computational approach used to determine the mechanism of protein-ligand interactions [53]. The docking between ATR and TYP was performed by using Autodock Vina to determine the binding sites and the binding mode of ATR on TYP [54]. The docked structure of the TYP-ATR system is given in Figure 7A–C. The ATR binds with TYP with the free binding energy of −4.1 Kcal mol^−1^. In the binding pocket of TYP, the ATR molecule was surrounded by amino acid residues (Figure 8A,B) Ala-221, Ser-190, Trp-215, Ser-214, Asp-189, Gly-226, Ser-195, Val-227, Gly-216, and Ser-217. The ATR also interacted with CYS-220 and Gly-219 with hydrogen bonds. Further, the His-57 and Cys-191 also interacted with ATR through pi-alkyl interaction. Moreover, the conformation changes caused by ATR binding with binding pockets interact directly with catalytic amino acid residues of the TYP. The results obtained from the docking agreed with our conclusion in the experiments.

#### 3.2.8. Effect of ATR on TYP Activity

**Figure 9:**
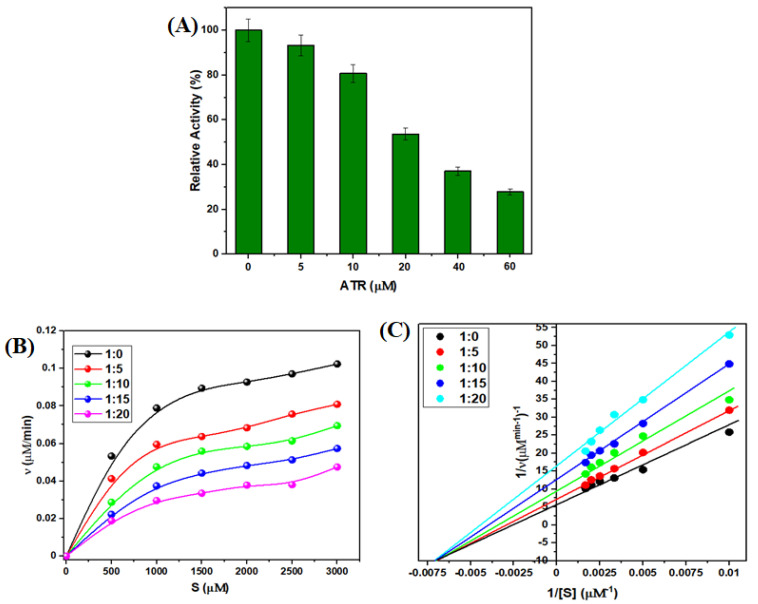
(**A**) Relative activity of TYP in the presence of ATR. (**B**) Michaelis-Menten curves of TYP with different concentration of ATR. (**C**) Double-reciprocal plots of TYP with different concentrations of ATR.

The spectroscopic and docking experiment results suggest an interaction between ATR and TYP. The relative activity (RA%) of the TYP was evaluated at different concentrations of ATR, and it was observed that the TYP activity decreased with the addition of ATR (Figure 9A). The TYP has three primary catalytic triads (His-57, Asp-102, and Ser-195) and a substrate-binding pocket (Asp-194, Gly-217, and Gly-227) which play a vital role in the binding of substrate to TYP [55,56]. The molecular docking confirmed that ATR interacts with the catalytic triad (His-57) and hence affects the enzymatic activity TYP. Furthermore, these results affirm that ATR interaction may lead to conformational changes in the structure of TYP, reducing its activity.

However, Michaelis-Menten curves (Figure 9B) of TYP suggest a decrease in the enzymatic activity of TYP in the presence of ATR. Additionally, the activity declined further with an increase in ATR concentration. The decreased enzymatic activity is attributed to conformational structure change and the catalytic ability of TYP [57]. The Lineweaver-Burk plots of TYP and TYP-ATR also confirmed the inhibition mode of ATR (Figure 9C). Moreover, it is clear from Table 4 that the V_max_ value decreased while the K_m_ values almost remained unchanged on interaction with higher concentrations of ATR. Further, the TYP-ATR complex formation shows a non-competitive inhibition. In addition, the catalytic constant k_cat_ and k_cat_/K_m_ values decreased gradually at higher concentrations of ATR [57]. Thus, the results suggest that ATR inhibits the enzymatic activity of TYP in a concentration-dependent manner.

## 4. Conclusions

This study provides a detailed binding mechanism of the TYP-ATR system and its effects on the structure conformations and the influence of these conformational changes on the enzymatic activities of the TYP. The results suggest a moderate binding interaction between TYP and ATR, and the binding strength was temperature-dependent. The molecular docking and thermodynamic results suggest the involvement of hydrophobic interaction and hydrogen bonds between TYP and ATR and a single binding site for ATR on TYP. In the TYP-ATR system, the TYP activity was inhibited with ATR in a concentration-dependent manner since ATR was bound to the catalytic site. This study provides a deeper understanding of the mechanism of binding of ATR with the digestive proteases from the point of view of toxicological effects.

## Figures and Tables

**Table 1:** The values of Stern-Volmer constant and quenching rate constant for the TYP-ATR system.

pH	Temp (K)	K_sv_ (×10^3^ M^−1^)	k_q_ (×10^11^ M^−1^ s^−1^)	R^2^
7.4	295	4.24	4.24	0.998
	305	3.28	3.28	0.996
	315	2.49	2.89	0.990

**Table 2:** The values of the binding constant and the number of binding sites for the interaction of ATR with TYP.

pH	Temp (K)	K_b_ (×10^3^ M^−1^)	n	R^2^
7.4	295	2.192	0.93	0.995
	305	1.974	0.95	0.980
	315	1.887	0.98	0.996

**Table 3:** Various thermodynamic parameters for TYP-ATR complex formation at various temperatures.

Temp (K)	ΔH (KJ mol^−1^)	ΔS (Jmol^−1^ k^−1^)	ΔG (KJ mol^−1^)	R^2^
295	5.8	44.21	−7.2	0.9954
305			−7.6	0.9988
315			−8.1	0.9986

**Table 4:** Michaelis-Menten kinetic parameters of TYP in the presence of ATR concentrations.

TYP/ATR	V_max_ (µM s^−1^)	K_m_ (µM)	K_cat_ (s^−1^)	K_cat_/K_m_ (µM^−1^s^−1^)
1:0	5.44 × 10^−4^	3.89 × 10^−3^	3.62 × 10^−5^	9.33 × 10^−3^
1:5	4.05 × 10^−4^	3.02 × 10^−3^	2.70 × 10^−5^	8.93 × 10^−3^
1:10	4.14 × 10^−4^	4.71 × 10^−3^	2.76 × 10^−5^	5.84 × 10^−3^
1:15	3.08 × 10^−4^	3.81 × 10^−3^	2.05 × 10^−5^	5.40 × 10^−3^
1:20	2.59 × 10^−4^	4.36 × 10^−3^	1.72 × 10^−5^	3.96 × 10^−3^

## Data Availability

Data will be available on request to corresponding author.

## References

[1] Gianessi L.P. (2013). The increasing importance of herbicides in worldwide crop production. Pest Manag. Sci..

[2] Kudsk P., Streibig J. (2003). Herbicides-a two-edged sword. Weed Res..

[3] Gupta P.K. (2018). Toxicity of Herbicides, Veterinary Toxicology.

[4] Mesnage R., Bernay B., Séralini G.-E. (2013). Ethoxylated adjuvants of glyphosate-based herbicides are active principles of human cell toxicity. Toxicology.

[5] Marin-Morales M.A., Ventura-Camargo B.D.C., Hoshina M.M. (2013). Toxicity of herbicides: Impact on aquatic and soil biota and human health. Herbicides-Current Research and Case Studies in Use.

[6] Ahmad A., Zafar A., Zargar S., Bazgaifan A., Wani T.A., Ahmad M. (2021). Protective effects of apigenin against edifenphos-induced genotoxicity and cytotoxicity in rat hepatocytes. J. Biomol. Struct. Dyn..

[7] Alavanja M. (2009). Pesticides use and exposure extensive worldwide. Rev. Environ. Health.

[8] Williams M., Boerboom C.M., Rabaey T.L. (2010). Significance of Atrazine in Sweet Corn Weed Management Systems. Weed Technol..

[9] Buchanan G.A., Hiltbold A.E. (1973). Performance and Persistence of Atrazine. Weed Sci..

[10] Comber S.D.W. (1999). Abiotic persistence of atrazine and simazine in water. Pestic. Sci..

[11] Schwab A.P., Splichal P.A., Banks M.K. (2006). Persistence of Atrazine and Alachlor in Ground Water Aquifers and Soil. Water Air Soil Pollut..

[12] Pathak R.K., Dikshit A.K. (2011). Atrazine and human health. Int. J. Ecosyst..

[13] Giusi G., Facciolo R.M., Canonaco M., Alleva E., Belloni V., Dessi’-Fulgheri F., Santucci D. (2006). The endocrine disruptor atrazine accounts for a dimorphic somatostatinergic neuronal expression pattern in mice. Toxicol. Sci..

[14] Hayes T. (2009). More feedback on whether atrazine is a potent endocrine disruptor chemical. Environ. Sci. Technol.-Columb..

[15] Eldridge J.C., Stevens J.T., Breckenridge C.B. (2008). Atrazine interaction with estrogen expression systems. Rev. Environ. Contam. Toxicol..

[16] McMullin T.S., Andersen M.E., Nagahara A., Lund T.D., Pak T., Handa R.J., Hanneman W.H. (2004). Evidence that atrazine and diaminochlorotriazine inhibit the estrogen/progesterone induced surge of luteinizing hormone in female Sprague-Dawley rats without changing estrogen receptor action. Toxicol. Sci..

[17] Xing H., Wang C., Wu H., Chen D., Li S., Xu S. (2015). Effects of atrazine and chlorpyrifos on DNA methylation in the brain and gonad of the common carp. Comp. Biochem. Physiol. Part C Toxicol. Pharmacol..

[18] Wang F., Yang Q.-W., Zhao W.-J., Du Q.-Y., Chang Z.-J. (2019). Effects of short-time exposure to atrazine on miRNA expression profiles in the gonad of common carp (Cyprinus carpio). BMC Genom..

[19] Karami-Mohajeri S., Abdollahi M. (2011). Toxic influence of organophosphate, carbamate, and organochlorine pesticides on cellular metabolism of lipids, proteins, and carbohydrates. Hum. Exp. Toxicol..

[20] Sharma A.K., Gaur K., Tiwari R.K., Gaur M.S. (2011). Computational interaction analysis of organophosphorus pesticides with different metabolic proteins in humans. J. Biomed. Res..

[21] Wani T.A., Alsaif N., Bakheit A.H., Zargar S., Al-Mehizia A.A., Khan A.A. (2020). Interaction of an abiraterone with calf thymus DNA: Investigation with spectroscopic technique and modelling studies. Bioorg. Chem..

[22] Shah D., Mital K. (2018). The Role of Trypsin:Chymotrypsin in Tissue Repair. Adv. Ther..

[23] Wang Y., Luo W., Reiser G. (2008). Reiser, Trypsin and trypsin-like proteases in the brain: Proteolysis and cellular functions. Cell. Mol. Life Sci..

[24] Whitcomb D.C., Lowe M.E. (2007). Human pancreatic digestive enzymes. Dig. Dis. Sci..

[25] Pillai J.R., Wali A.F., Menezes G.A., Rehman M.U., Wani T.A., Arafah A., Zargar S., Mir T.M. (2022). Chemical Composition Analysis, Cytotoxic, Antimicrobial and Antioxidant Activities of Physalis angulata L. A Comparative Study of Leaves and Fruit. Molecules.

[26] Liu Y., Cao R., Qin P., Liu R. (2012). Assessing the potential toxic effect of one persistent organic pollutant: Non-covalent interaction of dicofol with the enzyme trypsin. Spectrochim. Acta Part A Mol. Biomol. Spectrosc..

[27] Li X., Huo M., Zhao L., Cao Z., Xu M., Wan J., Niu Q., Huo C., Tang J., Liu R. (2020). Study of the effects of ultrafine carbon black on the structure and function of trypsin. J. Mol. Recognit..

[28] Xiao Q., Liang J., Luo H., Li H., Yang J., Huang S. (2020). Investigations of conformational structures and activities of trypsin and pepsin affected by food colourant allura red. J. Mol. Liq..

[29] Huang S., Li H., Liu Y., Yang L., Wang D., Xiao Q. (2020). Investigations of conformational structure and enzymatic activity of trypsin after its binding interaction with graphene oxide. J. Hazard. Mater..

[30] Harish B.S., Uppuluri K.B. (2018). Potential Anticoagulant Activity of Trypsin Inhibitor Purified from an Isolated Marine Bacterium Oceanimonas Sp. BPMS22 and its Kinetics. Mar. Biotechnol..

[31] Schmid F.X. (2001). Biological Macromolecules: UV-Visible Spectrophotometry.

[32] Anthis N.J., Clore G.M. (2013). Sequence-specific determination of protein and peptide concentrations by absorbance at 205 nm. Protein Sci..

[33] Wang Y.-Q., Zhang H.-M. (2014). Effects of Bisphenol S on the Structures and Activities of Trypsin and Pepsin. J. Agric. Food Chem..

[34] Wani T.A., Alsaif N.A., Alanazi M.M., Bakheit A.H., Khan A.A., Zargar S. (2021). Binding of colchicine and ascorbic acid (vitamin C) to bovine serum albumin: An in-vitro interaction study using multispectroscopic, molecular docking and molecular dynamics simulation study. J. Mol. Liq..

[35] Zargar S., Wani T.A. (2021). Exploring the binding mechanism and adverse toxic effects of persistent organic pollutant (dicofol) to human serum albumin: A biophysical, biochemical and computational approach. Chem.-Bio. Interact..

[36] Wani T.A., Bakheit A.H., Al-Majed A.A., Altwaijry N., Baquaysh A., Aljuraisy A., Zargar S. (2021). Binding and drug displacement study of colchicine and bovine serum albumin in presence of azithromycin using multispectroscopic techniques and molecular dynamic simulation. J. Mol. Liq..

[37] Lakowicz J.R. (2013). Principles of Fluorescence Spectroscopy.

[38] Zargar S., Wani T.A., Alsaif N.A., Khayyat A.I.A. (2022). A Comprehensive Investigation of Interactions between Antipsychotic Drug Quetiapine and Human Serum Albumin Using Multi-Spectroscopic, Biochemical, and Molecular Modeling Approaches. Molecules.

[39] Wani T.A., Alsaif N., Alanazi M.M., Bakheit A.H., Zargar S., Bhat M.A. (2021). A potential anticancer dihydropyrimidine derivative and its protein binding mechanism by multispectroscopic, molecular docking and molecular dynamic simulation along with its in-silico toxicity and metabolic profile. Eur. J. Pharm. Sci..

[40] Mokaberi P., Babayan-Mashhadi F., Zadeh Z.A.T., Saberi M.R., Chamani J. (2021). Analysis of the interaction behavior between Nano-Curcumin and two human serum proteins: Combining spectroscopy and molecular stimulation to understand protein-protein interaction. J. Biomol. Struct. Dyn..

[41] Alsaif N.A., Al-Mehizia A.A., Bakheit A.H., Zargar S., Wani T.A. (2020). A Spectroscopic, Thermodynamic and Molecular Docking Study of the Binding Mechanism of Dapoxetine with Calf Thymus DNA. S. Afr. J. Chem..

[42] Gerbanowski A., Rabiller C., Larré C., Guéguen J. (1999). Grafting of Aliphatic and Aromatic Probes on Bovine Serum Albumin: Influence on Its Structural and Physicochemical Characteristics. J. Protein Chem..

[43] Ross P.D., Subramanian S. (1981). Thermodynamics of protein association reactions: Forces contributing to stability. Biochemistry.

[44] Liu Y., Zhang G., Liao Y., Wang Y. (2015). Binding characteristics of psoralen with trypsin: Insights from spectroscopic and molecular modeling studies. Spectrochim. Acta Part A Mol. Biomol. Spectrosc..

[45] Lloyd J.B.F. (1971). Synchronized Excitation of Fluorescence Emission Spectra. Nat. Phys. Sci..

[46] Kou S.-B., Lin Z.-Y., Wang B.-L., Shi J.-H., Liu Y.-X. (2021). Evaluation of the binding behavior of olmutinib (HM61713) with model transport protein: Insights from spectroscopic and molecular docking studies. J. Mol. Struct..

[47] Ranjbar S., Shokoohinia Y., Ghobadi S., Bijari N., Gholamzadeh S., Moradi N., Ashrafi-Kooshk M.R., Aghaei A., Khodarahmi R. (2013). Studies of the Interaction between Isoimperatorin and Human Serum Albumin by Multispectroscopic Method: Identification of Possible Binding Site of the Compound Using Esterase Activity of the Protein. Sci. World, J..

[48] Wani T.A., Bakheit A.H., Zargar S., Khayyat A.I.A., Al-Majed A.A. (2022). Influence of Rutin, Sinapic Acid, and Naringenin on Binding of Tyrosine Kinase Inhibitor Erlotinib to Bovine Serum Albumin Using Analytical Techniques Along with Computational Approach. Appl. Sci..

[49] Johnson W.C. (1988). Secondary structure of proteins through circular dichroism spectroscopy. Annu. Rev. Biophys. Biophys. Chem..

[50] Sarkar D., Mahata A., Das P., Girigoswami A., Ghosh D., Chattopadhyay N. (2009). Deciphering the perturbation of serum albumins by a ketocyanine dye: A spectroscopic approach. J. Photochem. Photobiol. B Biol..

[51] Wani T.A., Bakheit A.H., Zargar S., Alanazi Z.S., Al-Majed A.A. (2020). Influence of antioxidant flavonoids quercetin and rutin on the in-vitro binding of neratinib to human serum albumin. Spectrochim. Acta Part A Mol. Biomol. Spectrosc..

[52] Darwish S.M., Abu Sharkh S.E., Abu Teir M.M., Makharza S., Abu-Hadid M.M. (2010). Spectroscopic investigations of pentobarbital interaction with human serum albumin. J. Mol. Struct..

[53] Zargar S., Wani T.A. (2021). Protective Role of Quercetin in Carbon Tetrachloride Induced Toxicity in Rat Brain: Biochemical, Spectrophotometric Assays and Computational Approach. Molecules.

[54] Trott O., Olson A.J. (2010). AutoDock Vina: Improving the speed and accuracy of docking with a new scoring function, efficient optimization, and multithreading. J. Comput. Chem..

[55] Higaki J., Gibson B., Craik C. (1987). Evolution of Catalysis in the Serine Proteases, Cold Spring Harbor Symposia on Quantitative Biology.

[56] Wang Y., Zhang H., Cao J., Zhou Q. (2013). Interaction of methotrexate with trypsin analyzed by spectroscopic and molecular modeling methods. J. Mol. Struct..

[57] Luo H., Li H., Liu Y., Yang L., Xiao Q., Huang S. (2021). Investigation on conformational variation and activity of trypsin affected by black phosphorus quantum dots via multi-spectroscopy and molecular modeling. Spectrochim. Acta Part A Mol. Biomol. Spectrosc..

